# Supplementary material for: Resistance to Combined Anthracycline–Taxane Chemotherapy Is Associated with Altered Metabolism and Inflammation in Breast Carcinomas
Source: Int J Mol Sci. 2024 Jan 15;25(2):1063. doi: 10.3390/ijms25021063 (PMC10816584; doi:10.3390/ijms25021063)
Supplement: Supplementary file 1 [file ijms-25-01063-s001.zip › Supplemental Table S1.pdf]

Supplemental Table S1:

Of the 224 overexpressed genes in tumor samples not developing PCR following an anthracycline-taxane treatment, 135 genes had AUC values of 0.7 or higher.

Gene	Mean-nonresponder	Mean-responder	Fold change	MannW_p	ROC AUC	ROC_AUC_p
**ORAI3**	934.6	580.6	1.61	7.87E-28	0.77	p<1E-16
**BCAM**	972.1	417.3	2.33	4.29E-25	0.756	p<1E-16
**ATP6V0A1**	945.1	592.5	1.6	9.53E-25	0.754	p<1E-16
**KIAA0556**	949.5	614.4	1.55	1.67E-24	0.753	p<1E-16
**TNK1**	833.5	463.8	1.8	5.87E-24	0.75	p<1E-16
**RAB26**	582.0	330.2	1.76	5.20E-24	0.75	p<1E-16
**ILK**	1440.0	964.5	1.49	1.59E-23	0.747	p<1E-16
**KIAA0586**	514.6	328.2	1.57	3.74E-23	0.745	p<1E-16
**FAM63A**	1178.4	673.4	1.75	4.67E-23	0.744	p<1E-16
**SKAP1**	662.9	378.9	1.75	9.76E-23	0.743	p<1E-16
**C8orf30A**	1418.2	831.1	1.71	9.02E-23	0.743	p<1E-16
**BLOC1S1**	1384.2	884.7	1.56	7.79E-23	0.743	p<1E-16
**ZNF629**	764.7	511.3	1.5	1.29E-22	0.742	p<1E-16
**GRINA**	968.8	570.7	1.7	2.19E-22	0.741	p<1E-16
**ITPKC**	630.8	404.4	1.56	3.39E-22	0.74	p<1E-16
**SALL2**	750.8	439.2	1.71	4.95E-22	0.739	p<1E-16
**P4HTM**	1576.4	923.0	1.71	3.82E-22	0.739	p<1E-16
**CNR2**	536.9	256.3	2.1	7.57E-22	0.737	p<1E-16
**C19orf22**	4693.4	2569.9	1.83	7.98E-22	0.737	p<1E-16
**APBB3**	581.0	358.1	1.62	1.11E-21	0.737	p<1E-16
**STXBP2**	516.7	269.2	1.92	1.20E-21	0.736	p<1E-16
**ADAP1**	959.8	581.7	1.65	1.36E-21	0.736	p<1E-16
**PPP2R4**	1018.9	632.0	1.61	1.47E-21	0.736	p<1E-16
**RAB17**	603.0	338.4	1.78	2.06E-21	0.735	p<1E-16
**NAPA**	1117.6	642.8	1.74	3.27E-21	0.734	p<1E-16
**CNNM3**	734.3	445.8	1.65	2.57E-21	0.734	p<1E-16
**DALRD3**	1316.1	812.5	1.62	4.01E-21	0.733	p<1E-16
**CHST10**	580.7	376.5	1.54	4.90E-21	0.733	p<1E-16
**GRIK5**	514.1	267.2	1.92	6.70E-21	0.732	p<1E-16
**PCGF1**	602.0	348.5	1.73	7.50E-21	0.732	p<1E-16
**FXYD3**	1842.2	1156.2	1.59	7.49E-21	0.732	p<1E-16
**GDI1**	1945.2	1259.5	1.54	6.76E-21	0.732	p<1E-16
**ERAL1**	629.5	408.6	1.54	7.41E-21	0.732	p<1E-16
**CNTD2**	769.0	411.0	1.87	9.85E-21	0.731	p<1E-16
**TPCN1**	839.1	551.1	1.52	1.06E-20	0.731	p<1E-16

**PCGF2**	762.2	501.9	1.52	1.05E-20	0.731	p<1E-16
**ZYX**	1062.1	612.9	1.73	1.22E-20	0.73	p<1E-16
**SCAMP5**	539.7	317.8	1.7	1.60E-20	0.73	p<1E-16
**ABHD4**	1138.7	748.3	1.52	1.54E-20	0.73	p<1E-16
**MGRN1**	1064.1	724.1	1.47	1.46E-20	0.73	p<1E-16
**ZNF552**	969.9	497.9	1.95	2.34E-20	0.729	p<1E-16
**CALCOCO1**	917.0	544.4	1.68	2.34E-20	0.729	p<1E-16
**FLOT2**	1435.5	902.7	1.59	1.88E-20	0.729	p<1E-16
**FPGS**	610.0	384.2	1.59	1.91E-20	0.729	p<1E-16
**TBC1D10B**	1008.1	667.1	1.51	2.39E-20	0.729	p<1E-16
**DOLPP1**	876.5	592.2	1.48	2.03E-20	0.729	p<1E-16
**TCTN2**	1515.0	747.9	2.03	3.05E-20	0.728	p<1E-16
**KDM6B**	1097.6	603.5	1.82	2.63E-20	0.728	p<1E-16
**PTPN9**	564.5	358.7	1.57	2.71E-20	0.728	p<1E-16
**DGKA**	687.4	436.7	1.57	2.80E-20	0.728	p<1E-16
**C9orf7**	525.1	320.1	1.64	4.68E-20	0.727	p<1E-16
**FAM102A**	1379.4	885.2	1.56	4.67E-20	0.727	p<1E-16
**NECAB3**	661.5	428.0	1.55	4.00E-20	0.727	p<1E-16
**MLEC**	1824.7	1190.8	1.53	3.72E-20	0.727	p<1E-16
**ABCA2**	513.6	307.9	1.67	7.29E-20	0.726	p<1E-16
**ROGDI**	857.5	524.2	1.64	6.30E-20	0.726	p<1E-16
**MOSPD3**	517.3	323.2	1.6	7.02E-20	0.726	p<1E-16
**NUCB1**	1075.0	658.4	1.63	9.24E-20	0.725	p<1E-16
**PHLDA3**	710.9	457.8	1.55	9.90E-20	0.725	p<1E-16
**MAZ**	1196.7	802.5	1.49	7.60E-20	0.725	p<1E-16
**TAC3**	511.9	273.9	1.87	1.14E-19	0.724	p<1E-16
**CCDC9**	658.5	384.9	1.71	1.20E-19	0.724	p<1E-16
**MYL4**	572.7	295.0	1.94	2.52E-19	0.722	p<1E-16
**WFS1**	1021.1	662.1	1.54	3.17E-19	0.722	p<1E-16
**PRR5**	736.6	508.7	1.45	2.78E-19	0.722	p<1E-16
**NR1D1**	978.7	540.1	1.81	3.30E-19	0.721	p<1E-16
**SLC2A9**	591.1	346.5	1.71	4.73E-19	0.721	p<1E-16
**IDUA**	501.7	309.5	1.62	3.68E-19	0.721	p<1E-16
**AGRN**	1091.5	685.8	1.59	5.09E-19	0.72	p<1E-16
**CHPF**	735.7	471.5	1.56	5.42E-19	0.72	p<1E-16
**BAP1**	640.3	440.0	1.46	5.50E-19	0.72	p<1E-16
**C19orf21**	589.1	292.4	2.02	9.43E-19	0.719	p<1E-16
**EPB49**	529.6	288.5	1.84	9.45E-19	0.719	p<1E-16
**ARFIP2**	1327.2	823.7	1.61	8.14E-19	0.719	p<1E-16
**ZNF467**	617.6	407.3	1.52	7.26E-19	0.719	p<1E-16
**MCRS1**	593.8	406.0	1.46	7.05E-19	0.719	p<1E-16
**PARP16**	553.4	382.0	1.45	7.89E-19	0.719	p<1E-16
**SAP25**	725.7	422.1	1.72	1.30E-18	0.718	p<1E-16

**RAB3IL1**	600.2	349.8	1.72	1.20E-18	0.718	p<1E-16
**IMPDH1**	965.1	655.1	1.47	1.06E-18	0.718	p<1E-16
**LOC171220**	736.7	472.7	1.56	1.61E-18	0.717	p<1E-16
**SORBS3**	545.7	357.9	1.52	1.88E-18	0.717	p<1E-16
**SHROOM2**	507.3	340.5	1.49	1.50E-18	0.717	p<1E-16
**TRMT2B**	564.2	350.3	1.61	2.75E-18	0.716	p<1E-16
**PPP2R1A**	1344.2	850.2	1.58	2.08E-18	0.716	p<1E-16
**MVK**	1301.6	823.5	1.58	2.68E-18	0.716	p<1E-16
**GIT1**	686.8	382.1	1.8	3.46E-18	0.715	p<1E-16
**IKBKG**	1010.3	589.0	1.72	2.97E-18	0.715	p<1E-16
**MID1IP1**	669.9	439.7	1.52	3.47E-18	0.715	p<1E-16
**SPR**	981.2	653.3	1.5	4.85E-18	0.714	p<1E-16
**OPHN1**	10424.7	5011.5	2.08	6.15E-18	0.713	p<1E-16
**GSK3A**	846.2	544.0	1.56	7.90E-18	0.713	p<1E-16
**AVPI1**	769.8	523.5	1.47	6.12E-18	0.713	p<1E-16
**GNAI2**	895.3	612.7	1.46	7.18E-18	0.713	p<1E-16
**REEP1**	667.0	369.0	1.81	9.62E-18	0.712	p<1E-16
**DMWD**	1034.8	609.9	1.7	1.07E-17	0.712	p<1E-16
**PXN**	930.2	631.0	1.47	9.76E-18	0.712	p<1E-16
**COX10**	751.3	513.4	1.46	9.45E-18	0.712	p<1E-16
**EEF1A2**	896.7	276.4	3.24	1.67E-17	0.711	p<1E-16
**EPHX1**	1378.9	728.5	1.89	1.22E-17	0.711	p<1E-16
**EML3**	619.5	405.2	1.53	1.43E-17	0.711	p<1E-16
**RMND5B**	619.5	422.8	1.47	1.58E-17	0.711	p<1E-16
**CGA**	823.3	212.8	3.87	1.71E-17	0.71	p<1E-16
**RNF122**	594.5	347.1	1.71	2.09E-17	0.71	p<1E-16
**MAP1A**	637.1	398.1	1.6	1.79E-17	0.71	p<1E-16
**BSG**	1157.0	767.1	1.51	2.09E-17	0.71	p<1E-16
**ARHGEF16**	514.1	341.5	1.51	2.38E-17	0.71	p<1E-16
**SLC22A5**	716.4	481.7	1.49	1.91E-17	0.71	p<1E-16
**SYT1**	712.5	404.9	1.76	3.16E-17	0.709	p<1E-16
**ARHGDIA**	2101.6	1335.1	1.57	2.69E-17	0.709	p<1E-16
**SAPS1**	595.7	407.0	1.46	3.23E-17	0.709	p<1E-16
**MEFV**	538.8	233.6	2.31	4.64E-17	0.708	p<1E-16
**AATK**	505.5	319.5	1.58	3.99E-17	0.708	p<1E-16
**KIAA1467**	907.3	536.6	1.69	4.94E-17	0.707	p<1E-16
**ATP10B**	572.4	318.7	1.8	8.06E-17	0.706	p<1E-16
**BANF1**	2731.5	1748.7	1.56	8.49E-17	0.706	p<1E-16
**TSPYL2**	622.4	402.3	1.55	7.62E-17	0.706	p<1E-16
**C12orf49**	753.9	510.1	1.48	9.29E-17	0.706	p<1E-16
**IPO13**	714.4	483.7	1.48	6.74E-17	0.706	p<1E-16
**UBA1**	3649.4	2508.7	1.45	7.65E-17	0.706	p<1E-16
**SEMA4A**	744.7	474.0	1.57	1.12E-16	0.705	p<1E-16

**REEP4**	876.1	599.7	1.46	1.07E-16	0.705	p<1E-16
**CYP1A2**	524.9	297.9	1.76	1.37E-16	0.704	p<1E-16
**CYP3A4**	593.2	352.3	1.68	1.64E-16	0.704	p<1E-16
**CLPTM1**	690.1	422.3	1.63	1.47E-16	0.704	p<1E-16
**PCBD1**	772.0	499.3	1.55	1.62E-16	0.704	p<1E-16
**PKM2**	2953.3	1768.5	1.67	2.28E-16	0.703	p<1E-16
**OGFR**	695.9	464.1	1.5	2.40E-16	0.703	p<1E-16
**CEP250**	586.2	400.1	1.47	2.29E-16	0.703	p<1E-16
**SPATA20**	1134.0	747.5	1.52	3.23E-16	0.702	p<1E-16
**MIIP**	981.6	670.3	1.46	3.35E-16	0.702	p<1E-16
**TRIP10**	554.4	352.4	1.57	3.72E-16	0.701	p<1E-16
**FMO4**	642.5	434.9	1.48	5.04E-16	0.701	p<1E-16
**PPP2R5D**	1200.6	821.0	1.46	4.48E-16	0.701	p<1E-16
**CYP2A6**	667.5	241.7	2.76	6.73E-16	0.7	p<1E-16
