# Supplementary material for: Resistance to Combined Anthracycline–Taxane Chemotherapy Is Associated with Altered Metabolism and Inflammation in Breast Carcinomas
Source: Int J Mol Sci. 2024 Jan 15;25(2):1063. doi: 10.3390/ijms25021063 (PMC10816584; doi:10.3390/ijms25021063)
Supplement: Supplementary file 1 [file ijms-25-01063-s001.zip › Supplemental Table S2.pdf]

**Supplemental Table S2**

**The 51 genes differentiating responders and nonresponders in the RFS dataset and their performance in the PCR dataset**

Only six genes, labeled with green font and highlighted, were significant in both datasets. For the rest of the genes in the PCR dataset, the association was either nonsignificant or the direction was reversed (Fold Change < 1); thus, gene expression was higher in responders.

| | RFS | | | | | | PCR | | |
|---|---|---|---|---|---|---|---|---|---|
| GENE SYMBOL | N_nonresp | N_resp | Fold Change | MannW_p | ROC AUC | ROC_AUC_p | Fold Change | ROC AUC | ROC_AUC_p |
| KMO | 121 | 66 | 1.45 | 0.00011447 | 0.671 | 2.70E-05 | 1.08 | 0.585 | 0.00029 |
| UBE2D1 | 121 | 66 | 1.45 | 1.83E-05 | 0.69 | 1.20E-06 | 0.63 | 0.698 | 0 |
| VSIG4 | 121 | 66 | 1.45 | 8.50E-05 | 0.674 | 1.20E-05 | 0.89 | 0.533 | 0.094 |
| SLA | 121 | 66 | 1.45 | 0.00010742 | 0.672 | 8.60E-06 | 1.11 | 0.521 | 0.19 |
| CPVL | 121 | 66 | 1.46 | 2.40E-05 | 0.687 | 4.00E-06 | 0.87 | 0.588 | 0.00011 |
| DOK5 | 121 | 66 | 1.46 | 4.09E-05 | 0.682 | 4.80E-06 | 1.03 | 0.538 | 0.063 |
| RGL1 | 121 | 66 | 1.47 | 2.02E-06 | 0.71 | 5.10E-08 | 0.85 | 0.584 | 0.0002 |
| LPXN | 121 | 66 | 1.47 | 1.58E-05 | 0.691 | 5.00E-07 | 1.03 | 0.502 | 0.48 |
| HEY1 | 121 | 66 | 1.47 | 5.92E-05 | 0.678 | 7.00E-06 | 1.03 | 0.528 | 0.13 |
| C11orf75 | 121 | 66 | 1.48 | 2.67E-05 | 0.686 | 6.60E-06 | 0.73 | 0.669 | 9.2E-15 |
| SEMA4D | 121 | 66 | 1.48 | 6.55E-05 | 0.677 | 3.50E-06 | 1.07 | 0.512 | 0.31 |
| C1orf38 | 121 | 66 | 1.49 | 2.19E-06 | 0.71 | 1.00E-07 | 1.20 | 0.538 | 0.055 |
| SLC9A6 | 121 | 66 | 1.49 | 1.28E-05 | 0.693 | 2.10E-06 | 0.90 | 0.59 | 0.000052 |
| SAT1 | 121 | 66 | 1.49 | 5.19E-05 | 0.679 | 6.80E-06 | 0.67 | 0.674 | 5.4E-14 |
| TUBB6 | 121 | 66 | 1.49 | 8.45E-05 | 0.674 | 1.30E-05 | 0.69 | 0.695 | 0 |
| PRAME | 121 | 66 | 1.50 | 1.43E-05 | 0.692 | 1.50E-06 | 1.03 | 0.55 | 0.029 |
| SDCBP | 121 | 66 | 1.50 | 1.03E-05 | 0.695 | 1.90E-07 | 0.81 | 0.634 | 6.1E-09 |
| ENPP2 | 121 | 66 | 1.51 | 4.62E-05 | 0.681 | 1.10E-05 | 0.59 | 0.673 | 3.6E-14 |
| CD14 | 121 | 66 | 1.51 | 0.00013297 | 0.669 | 4.10E-05 | 0.90 | 0.603 | 0.000006 |
| AIM2 | 121 | 66 | 1.52 | 0.00012846 | 0.67 | 1.60E-05 | 1.06 | 0.552 | 0.023 |
| MX2 | 121 | 66 | 1.52 | 9.48E-06 | 0.696 | 5.70E-07 | 1.12 | 0.537 | 0.062 |
| DTX4 | 121 | 66 | 1.52 | 0.00013528 | 0.669 | 2.10E-05 | 1.05 | 0.537 | 0.07 |
| PLAC8 | 121 | 66 | 1.54 | 0.0001192 | 0.67 | 1.70E-05 | 1.19 | 0.559 | 0.0095 |
| DFNA5 | 121 | 66 | 1.54 | 1.97E-07 | 0.73 | 7.10E-10 | 0.97 | 0.537 | 0.063 |
| LBH | 121 | 66 | 1.55 | 1.94E-05 | 0.689 | 1.00E-06 | 0.83 | 0.595 | 0.000037 |
| MSN | 121 | 66 | 1.56 | 3.67E-05 | 0.683 | 5.80E-06 | 0.60 | 0.71 | 0 |
| WIPF1 | 121 | 66 | 1.59 | 7.12E-05 | 0.676 | 9.30E-06 | 0.62 | 0.67 | 3.4E-13 |

| | | | | | | | | | |
|---|---|---|---|---|---|---|---|---|---|
| NXN | 121 | 66 | 1.62 | 1.22E-05 | 0.694 | 1.20E-06 | 0.78 | 0.646 | 5.2E-11 |
| HCLS1 | 121 | 66 | 1.63 | 0.00012059 | 0.67 | 1.10E-05 | 1.00 | 0.527 | 0.13 |
| **VSNL1** | **121** | **66** | **1.66** | **2.95E-05** | **0.685** | **2.10E-06** | **1.24** | **0.583** | **0.00045** |
| GABRE | 121 | 66 | 1.66 | 8.99E-06 | 0.697 | 5.90E-07 | 0.89 | 0.505 | 0.42 |
| PLS3 | 121 | 66 | 1.68 | 6.91E-06 | 0.699 | 6.10E-07 | 0.60 | 0.701 | 0 |
| SEPT6 | 121 | 66 | 1.68 | 8.15E-06 | 0.698 | 3.10E-07 | 0.80 | 0.625 | 0.000000076 |
| GPNMB | 121 | 66 | 1.69 | 9.11E-06 | 0.697 | 5.40E-07 | 0.70 | 0.672 | 1.4E-14 |
| CCR1 | 121 | 66 | 1.70 | 7.14E-07 | 0.72 | 6.80E-09 | 1.00 | 0.535 | 0.077 |
| LMO2 | 121 | 66 | 1.71 | 6.50E-07 | 0.72 | 2.60E-09 | 1.05 | 0.507 | 0.39 |
| ANXA1 | 121 | 66 | 1.72 | 5.41E-07 | 0.722 | 1.10E-08 | 0.75 | 0.654 | 4.6E-12 |
| SLC7A5 | 121 | 66 | 1.72 | 3.01E-05 | 0.685 | 6.30E-06 | 0.89 | 0.561 | 0.0062 |
| ACOT9 | 121 | 66 | 1.74 | 2.71E-07 | 0.728 | 3.80E-10 | 0.82 | 0.628 | 7.1E-09 |
| ADM | 121 | 66 | 1.76 | 7.08E-05 | 0.676 | 7.00E-06 | 0.82 | 0.581 | 0.00031 |
| PLOD2 | 121 | 66 | 1.79 | 3.54E-06 | 0.705 | 9.10E-08 | 0.64 | 0.69 | 0 |
| FOLR1 | 121 | 66 | 1.84 | 3.36E-05 | 0.684 | 4.20E-06 | 0.98 | 0.546 | 0.042 |
| EVI2A | 121 | 66 | 1.84 | 6.40E-05 | 0.677 | 4.10E-06 | 1.03 | 0.535 | 0.074 |
| HMOX1 | 121 | 66 | 1.86 | 7.50E-08 | 0.738 | 4.80E-11 | 0.87 | 0.574 | 0.0013 |
| LYN | 121 | 66 | 1.88 | 1.08E-08 | 0.753 | 3.50E-12 | 0.73 | 0.653 | 1.2E-11 |
| CHI3L1 | 121 | 66 | 2.03 | 3.53E-05 | 0.683 | 7.40E-06 | 0.71 | 0.611 | 0.0000011 |
| LY96 | 121 | 66 | 2.04 | 9.71E-08 | 0.736 | 6.30E-11 | 0.78 | 0.63 | 0.000000013 |
| RARRES1 | 121 | 66 | 2.22 | 2.76E-05 | 0.686 | 3.00E-06 | 0.76 | 0.677 | 3.3E-16 |
| TMEM45A | 121 | 66 | 2.35 | 8.21E-05 | 0.674 | 7.00E-06 | 0.81 | 0.637 | 7E-10 |
| **CALB2** | **121** | **66** | **2.39** | **7.57E-07** | **0.719** | **9.70E-09** | **1.08** | **0.566** | **0.0051** |
| DEFB1 | 121 | 66 | 2.66 | 7.47E-05 | 0.675 | 9.80E-06 | 1.07 | 0.51 | 0.35 |
