# Supplementary material for: Resistance to Combined Anthracycline–Taxane Chemotherapy Is Associated with Altered Metabolism and Inflammation in Breast Carcinomas
Source: Int J Mol Sci. 2024 Jan 15;25(2):1063. doi: 10.3390/ijms25021063 (PMC10816584; doi:10.3390/ijms25021063)
Supplement: Supplementary file 1 [file ijms-25-01063-s001.zip › Supplemental Table S3.pdf]

Supplemental Table S3

The 224 genes differentiating responders and nonresponders in the PCR dataset and their performance in the RFS dataset

Only two genes, labeled with green font and highlighted, were significant in both datasets. For the rest of the genes in the RFS dataset, the association was either nonsignificant or the direction was reversed (Fold Change < 1); thus, gene expression was higher in responders.

			**PCR**				**RFS**		
GENE SYMBOL	N_nonresp	N_resp	Fold Change	MannW_p	ROC AUC	ROC_AUC_p	Fold Change	ROC AUC	ROC_AUC_p
PRR5	435	199	1.45	2.78E-19	0.722	0	1.09	0.574	0.043
PARP16	435	199	1.45	7.89E-19	0.719	0	1.13	0.587	0.021
UBA1	435	199	1.45	7.65E-17	0.706	0	0.93	0.594	0.013
PNPLA6	435	199	1.45	7.2E-16	0.699	0	1.07	0.54	0.18
TESK2	435	199	1.45	1.89E-15	0.697	0	1.02	0.509	0.42
ALOX15B	435	199	1.45	3.49E-14	0.687	1.1E-16	0.42	0.519	0.34
ACTN4	435	199	1.45	1.1E-12	0.676	3.1E-13	1.05	0.537	0.19
EMP3	435	199	1.45	5.93E-10	0.653	2.1E-11	1.32	0.6	0.0087
BAP1	435	199	1.46	5.5E-19	0.72	0	0.98	0.524	0.3
MCRS1	435	199	1.46	7.05E-19	0.719	0	0.86	0.656	0.000094
GNAI2	435	199	1.46	7.18E-18	0.713	0	0.93	0.551	0.12
COX10	435	199	1.46	9.45E-18	0.712	0	1.08	0.566	0.066
SAPS1	435	199	1.46	3.23E-17	0.709	0	0.96	0.517	0.35
REEP4	435	199	1.46	1.07E-16	0.705	0	1.05	0.551	0.13
MIIP	435	199	1.46	3.35E-16	0.702	0	1.21	0.661	0.000037
PPP2R5D	435	199	1.46	4.48E-16	0.701	0	1.12	0.586	0.025
SREBF2	435	199	1.46	7.03E-15	0.692	1.1E-16	1.06	0.514	0.37
RAP1GAP	435	199	1.46	9.72E-13	0.676	1.5E-14	0.92	0.503	0.47
RHOT2	435	199	1.46	1.76E-11	0.666	4.6E-15	0.77	0.637	0.00068
PTGES	435	199	1.46	7.96E-08	0.633	9.6E-09	0.85	0.509	0.43
MGRN1	435	199	1.47	1.46E-20	0.73	0	0.92	0.586	0.027
IMPDH1	435	199	1.47	1.06E-18	0.718	0	1.11	0.574	0.047
AVPI1	435	199	1.47	6.12E-18	0.713	0	1.21	0.634	0.0005
PXN	435	199	1.47	9.76E-18	0.712	0	1.09	0.575	0.047
RMND5B	435	199	1.47	1.58E-17	0.711	0	0.91	0.607	0.0087
CEP250	435	199	1.47	2.29E-16	0.703	0	1.07	0.546	0.15

EML2	435	199	1.47	2.8E-15	0.695	0	0.86	0.587	0.027
ITGA5	435	199	1.47	1.02E-12	0.676	6.9E-15	1.20	0.6	0.01
S100A14	435	199	1.47	2.23E-08	0.638	2.4E-09	0.94	0.546	0.15
DOLPP1	435	199	1.48	2.03E-20	0.729	0	1.16	0.641	0.00064
IPO13	435	199	1.48	6.74E-17	0.706	0	1.12	0.601	0.0087
C12orf49	435	199	1.48	9.29E-17	0.706	0	1.32	0.699	0.00000017
FMO4	435	199	1.48	5.04E-16	0.701	0	1.31	0.672	0.000014
B4GALT2	435	199	1.48	1.18E-15	0.698	0	1.09	0.531	0.24
CIDEB	435	199	1.48	1.71E-14	0.69	4.4E-16	1.21	0.639	0.00054
MCCC2	435	199	1.48	4.87E-13	0.679	2.3E-15	0.66	0.642	0.00084
ILK	435	199	1.49	1.59E-23	0.747	0	0.94	0.554	0.12
MAZ	435	199	1.49	7.6E-20	0.725	0	0.94	0.567	0.063
SHROOM2	435	199	1.49	1.5E-18	0.717	0	0.97	0.513	0.39
SLC22A5	435	199	1.49	1.91E-17	0.71	0	0.95	0.517	0.36
SLC7A8	435	199	1.49	8.36E-14	0.685	0	0.73	0.612	0.0067
GLB1L2	435	199	1.49	2.38E-12	0.673	4.8E-13	1.02	0.504	0.47
SCCPDH	435	199	1.49	3.77E-12	0.672	1.9E-14	0.78	0.591	0.02
DHRS9	435	199	1.49	3.22E-10	0.655	2.9E-11	1.43	0.646	0.00013
RHOH	435	199	1.49	0.000000278	0.627	2.4E-08	0.72	0.62	0.0029
ZNF629	435	199	1.5	1.29E-22	0.742	0	0.95	0.551	0.13
SPR	435	199	1.5	4.85E-18	0.714	0	0.87	0.556	0.11
OGFR	435	199	1.5	2.4E-16	0.703	0	1.10	0.599	0.014
C11orf51	435	199	1.5	8.03E-16	0.699	0	0.96	0.551	0.13
TOP1	435	199	1.5	1.67E-14	0.69	2.2E-16	0.98	0.503	0.48
GPC1	435	199	1.5	1.06E-12	0.676	7.8E-16	0.78	0.602	0.012
SELP	435	199	1.5	2.79E-12	0.673	2.1E-14	1.24	0.661	0.000055
TBC1D10B	435	199	1.51	2.39E-20	0.729	0	1.10	0.589	0.021
BSG	435	199	1.51	2.09E-17	0.71	0	0.83	0.628	0.0012
ARHGEF16	435	199	1.51	2.38E-17	0.71	0	0.93	0.539	0.2
NELL2	435	199	1.51	0.000000526	0.624	0.00000012	0.72	0.537	0.21
MAGED2	435	199	1.51	0.00000347	0.615	0.00000038	0.57	0.688	0.0000016
PCGF2	435	199	1.52	1.05E-20	0.731	0	0.98	0.523	0.3
TPCN1	435	199	1.52	1.06E-20	0.731	0	0.92	0.539	0.2
ABHD4	435	199	1.52	1.54E-20	0.73	0	1.02	0.511	0.41
ZNF467	435	199	1.52	7.26E-19	0.719	0	1.03	0.51	0.42

SORBS3	435	199	1.52	1.88E-18	0.717	0	0.97	0.503	0.48
MID1IP1	435	199	1.52	3.47E-18	0.715	0	1.24	0.625	0.0014
SPATA20	435	199	1.52	3.23E-16	0.702	0	0.76	0.621	0.004
CEACAM6	435	199	1.52	7.21E-08	0.633	2.5E-08	0.92	0.557	0.099
MLEC	435	199	1.53	3.72E-20	0.727	0	0.91	0.594	0.017
EML3	435	199	1.53	1.43E-17	0.711	0	1.03	0.53	0.25
CA12	435	199	1.53	0.000000565	0.624	0.00000016	0.56	0.642	0.00075
CRABP2	435	199	1.53	0.000000867	0.622	0.00000013	0.81	0.546	0.15
CHST10	435	199	1.54	4.9E-21	0.733	0	1.07	0.538	0.19
GDI1	435	199	1.54	6.76E-21	0.732	0	0.91	0.52	0.33
ERAL1	435	199	1.54	7.41E-21	0.732	0	0.96	0.525	0.29
WFS1	435	199	1.54	3.17E-19	0.722	0	0.81	0.622	0.0033
GAS8	435	199	1.54	1.01E-15	0.698	0	1.06	0.504	0.46
NKX3-1	435	199	1.54	5.45E-13	0.678	6.8E-15	0.65	0.583	0.035
KIAA0556	435	199	1.55	1.67E-24	0.753	0	0.92	0.563	0.084
NECAB3	435	199	1.55	4E-20	0.727	0	0.87	0.564	0.076
PHLDA3	435	199	1.55	9.9E-20	0.725	0	0.99	0.526	0.29
TSPYL2	435	199	1.55	7.62E-17	0.706	0	0.93	0.528	0.26
PCBD1	435	199	1.55	1.62E-16	0.704	0	0.88	0.587	0.024
BLOC1S1	435	199	1.56	7.79E-23	0.743	0	0.86	0.617	0.0041
ITPKC	435	199	1.56	3.39E-22	0.74	0	1.09	0.555	0.1
FAM102A	435	199	1.56	4.67E-20	0.727	0	0.89	0.586	0.027
CHPF	435	199	1.56	5.42E-19	0.72	0	1.08	0.548	0.15
LOC171220	435	199	1.56	1.61E-18	0.717	0	1.11	0.581	0.032
GSK3A	435	199	1.56	7.9E-18	0.713	0	1.07	0.553	0.11
BANF1	435	199	1.56	8.49E-17	0.706	0	0.80	0.645	0.00022
KIAA0586	435	199	1.57	3.74E-23	0.745	0	1.04	0.533	0.23
PTPN9	435	199	1.57	2.71E-20	0.728	0	1.05	0.545	0.15
DGKA	435	199	1.57	2.8E-20	0.728	0	1.16	0.606	0.0061
ARHGDIA	435	199	1.57	2.69E-17	0.709	0	0.89	0.572	0.056
SEMA4A	435	199	1.57	1.12E-16	0.705	0	1.03	0.532	0.24
TRIP10	435	199	1.57	3.72E-16	0.701	0	1.11	0.569	0.063
RAB6B	435	199	1.57	1.35E-15	0.698	0	1.26	0.647	0.00021
THTPA	435	199	1.57	2.9E-15	0.695	0	0.92	0.552	0.12
SLC22A18	435	199	1.57	3.23E-15	0.695	0	0.79	0.613	0.0063

G6PD	435	199	1.57	3.09E-11	0.664	1.5E-12	0.99	0.572	0.052
CREB3L1	435	199	1.57	2.61E-10	0.656	2.9E-12	0.88	0.548	0.14
PPP2R1A	435	199	1.58	2.08E-18	0.716	0	0.82	0.697	0.00000036
MVK	435	199	1.58	2.68E-18	0.716	0	1.11	0.587	0.022
AATK	435	199	1.58	3.99E-17	0.708	0	1.03	0.539	0.2
IL2RG	435	199	1.58	1.4E-12	0.675	3.3E-13	1.26	0.645	0.00027
PON3	435	199	1.58	4.77E-08	0.635	6.1E-09	0.81	0.511	0.4
TTC39A	435	199	1.58	0.00000312	0.615	0.00000041	0.60	0.625	0.0028
FXYD3	435	199	1.59	7.49E-21	0.732	0	1.02	0.511	0.41
FLOT2	435	199	1.59	1.88E-20	0.729	0	1.03	0.537	0.2
FPGS	435	199	1.59	1.91E-20	0.729	0	1.10	0.561	0.081
AGRN	435	199	1.59	5.09E-19	0.72	0	1.03	0.528	0.27
GPSM3	435	199	1.59	2.21E-12	0.674	1.4E-14	1.23	0.605	0.0067
ATP6V0A1	435	199	1.6	9.53E-25	0.754	0	1.01	0.501	0.49
MOSPD3	435	199	1.6	7.02E-20	0.726	0	1.00	0.529	0.25
MAP1A	435	199	1.6	1.79E-17	0.71	0	1.23	0.633	0.00092
RASSF7	435	199	1.6	8.93E-12	0.669	4.7E-15	0.72	0.685	0.0000038
CRIP2	435	199	1.6	2.21E-11	0.665	1E-13	0.66	0.701	0.00000024
ORAI3	435	199	1.61	7.87E-28	0.77	0	0.95	0.527	0.28
PPP2R4	435	199	1.61	1.47E-21	0.736	0	0.86	0.652	0.00012
ARFIP2	435	199	1.61	8.14E-19	0.719	0	0.71	0.69	0.000005
TRMT2B	435	199	1.61	2.75E-18	0.716	0	1.29	0.685	0.0000012
NOVA1	435	199	1.61	4.56E-08	0.635	6.2E-09	0.72	0.547	0.16
CST3	435	199	1.61	0.00000254	0.616	0.00000016	0.68	0.668	0.000021
APBB3	435	199	1.62	1.11E-21	0.737	0	1.10	0.566	0.064
DALRD3	435	199	1.62	4.01E-21	0.733	0	0.81	0.606	0.01
IDUA	435	199	1.62	3.68E-19	0.721	0	0.78	0.622	0.0035
PRKCSH	435	199	1.62	1.64E-14	0.69	4.1E-15	0.87	0.615	0.0029
SLC9A3R1	435	199	1.62	9.98E-11	0.66	1.9E-12	0.69	0.669	0.000023
NUCB1	435	199	1.63	9.24E-20	0.725	0	0.85	0.597	0.013
CLPTM1	435	199	1.63	1.47E-16	0.704	0	0.77	0.663	0.000044
C9orf7	435	199	1.64	4.68E-20	0.727	0	0.87	0.599	0.014
ROGDI	435	199	1.64	6.3E-20	0.726	0	0.78	0.652	0.00035
FUT6	435	199	1.64	1.68E-14	0.69	2.2E-16	1.26	0.599	0.011
KRT18	435	199	1.64	6.55E-13	0.678	1.6E-15	0.79	0.635	0.00083

ESR1	435	199	1.64	1.11E-10	0.66	1.8E-11	0.44	0.611	0.0099
ADCY1	435	199	1.64	2.11E-09	0.648	3.5E-10	0.62	0.567	0.075
ADAP1	435	199	1.65	1.36E-21	0.736	0	1.08	0.563	0.082
CNNM3	435	199	1.65	2.57E-21	0.734	0	0.88	0.63	0.0016
ABCA3	435	199	1.65	8.29E-12	0.669	2.3E-14	0.66	0.679	0.00001
HOXC10	435	199	1.65	1.29E-09	0.65	2.5E-11	0.84	0.516	0.38
LYPD3	435	199	1.66	1.19E-13	0.683	4.4E-16	0.83	0.575	0.044
KIF5C	435	199	1.66	6.66E-13	0.678	7.5E-15	0.70	0.569	0.066
FAM174B	435	199	1.66	2.43E-12	0.673	1.6E-15	0.50	0.647	0.00028
ABCA2	435	199	1.67	7.29E-20	0.726	0	1.15	0.589	0.022
PKM2	435	199	1.67	2.28E-16	0.703	0	1.01	0.535	0.21
CALCOCO1	435	199	1.68	2.34E-20	0.729	0	1.10	0.547	0.14
CYP3A4	435	199	1.68	1.64E-16	0.704	0	1.25	0.616	0.0027
KIAA1467	435	199	1.69	4.94E-17	0.707	0	0.85	0.559	0.094
SPDEF	435	199	1.69	8.84E-15	0.692	0	0.62	0.672	0.000051
ZNF580	435	199	1.69	1.93E-08	0.639	2E-10	0.59	0.7	0.00000016
GRINA	435	199	1.7	2.19E-22	0.741	0	1.00	0.547	0.14
SCAMP5	435	199	1.7	1.6E-20	0.73	0	1.14	0.6	0.009
DMWD	435	199	1.7	1.07E-17	0.712	0	1.09	0.535	0.21
C8orf30A	435	199	1.71	9.02E-23	0.743	0	1.24	0.642	0.00037
P4HTM	435	199	1.71	3.82E-22	0.739	0	0.79	0.587	0.032
SALL2	435	199	1.71	4.95E-22	0.739	0	0.97	0.531	0.24
CCDC9	435	199	1.71	1.2E-19	0.724	0	1.14	0.567	0.057
SLC2A9	435	199	1.71	4.73E-19	0.721	0	1.13	0.6	0.011
RNF122	435	199	1.71	2.09E-17	0.71	0	1.14	0.601	0.01
NAT1	435	199	1.71	0.00000686	0.611	0.0000017	0.41	0.652	0.00023
RAB3IL1	435	199	1.72	1.2E-18	0.718	0	1.11	0.598	0.013
SAP25	435	199	1.72	1.3E-18	0.718	0	1.18	0.605	0.0073
IKBKG	435	199	1.72	2.97E-18	0.715	0	1.17	0.63	0.001
PCGF1	435	199	1.73	7.5E-21	0.732	0	0.92	0.57	0.055
ZYX	435	199	1.73	1.22E-20	0.73	0	1.11	0.583	0.032
HSPB1	435	199	1.73	0.000000203	0.628	1.4E-08	0.73	0.59	0.022
NAPA	435	199	1.74	3.27E-21	0.734	0	0.74	0.706	0.00000014
RHOB	435	199	1.74	6.31E-12	0.67	7.3E-15	0.58	0.671	0.000047
FAM63A	435	199	1.75	4.67E-23	0.744	0	0.76	0.63	0.0021

SKAP1	435	199	1.75	9.76E-23	0.743	0	1.06	0.537	0.2
RAB26	435	199	1.76	5.2E-24	0.75	0	0.93	0.548	0.14
SYT1	435	199	1.76	3.16E-17	0.709	0	0.97	0.51	0.41
CYP1A2	435	199	1.76	1.37E-16	0.704	0	1.30	0.597	0.0098
FBP1	435	199	1.76	2.03E-12	0.674	2.3E-15	0.62	0.672	0.000048
MLPH	435	199	1.76	2.39E-11	0.665	1.8E-13	0.48	0.68	0.000042
CEACAM5	435	199	1.77	1.45E-15	0.697	0	0.86	0.519	0.34
RAB17	435	199	1.78	2.06E-21	0.735	0	0.90	0.561	0.086
IGFBP4	435	199	1.78	7.28E-09	0.643	9.7E-11	0.61	0.64	0.00031
TNK1	435	199	1.8	5.87E-24	0.75	0	1.09	0.58	0.036
GIT1	435	199	1.8	3.46E-18	0.715	0	1.11	0.53	0.25
ATP10B	435	199	1.8	8.06E-17	0.706	0	1.22	0.621	0.0023
NR1D1	435	199	1.81	3.3E-19	0.721	0	1.24	0.601	0.0081
REEP1	435	199	1.81	9.62E-18	0.712	0	0.76	0.534	0.24
FPR1	435	199	1.81	0.00000308	0.615	0.00000041	1.61	0.568	0.063
KDM6B	435	199	1.82	2.63E-20	0.728	0	0.99	0.501	0.49
C19orf22	435	199	1.83	7.98E-22	0.737	0	1.14	0.55	0.12
EPB49	435	199	1.84	9.45E-19	0.719	0	1.15	0.55	0.12
TAT	435	199	1.84	4.24E-12	0.671	1.3E-13	0.44	0.573	0.063
SLC44A4	435	199	1.85	4.68E-11	0.663	3.7E-13	0.49	0.667	0.000066
FASN	435	199	1.86	4.13E-11	0.663	4.6E-13	0.55	0.649	0.0003
CNTD2	435	199	1.87	9.85E-21	0.731	0	1.03	0.517	0.35
TAC3	435	199	1.87	1.14E-19	0.724	0	1.10	0.576	0.046
ECM1	435	199	1.87	3.09E-14	0.688	1.1E-16	0.99	0.524	0.31
PF4	435	199	1.88	2.36E-12	0.673	1.2E-13	1.59	0.521	0.31
EPHX1	435	199	1.89	1.22E-17	0.711	0	0.78	0.615	0.0038
RAMP3	435	199	1.89	2.32E-13	0.681	2.2E-16	0.96	0.538	0.21
HBB	435	199	1.9	5.21E-09	0.644	9.3E-11	0.85	0.501	0.49
STXBP2	435	199	1.92	1.2E-21	0.736	0	0.72	0.666	0.000061
GRIK5	435	199	1.92	6.7E-21	0.732	0	1.06	0.557	0.11
CDC5L	435	199	1.93	2.17E-15	0.696	0	1.25	0.585	0.024
MYL4	435	199	1.94	2.52E-19	0.722	0	1.39	0.612	0.0048
ZNF552	435	199	1.95	2.34E-20	0.729	0	0.86	0.555	0.11
FOXA1	435	199	1.98	1.46E-15	0.697	0	0.52	0.685	0.00003
C19orf21	435	199	2.02	9.43E-19	0.719	0	0.85	0.555	0.12

CYP2B7P1	435	199	2.02	1.14E-11	0.668	9.6E-14	0.31	0.635	0.0011
TCTN2	435	199	2.03	3.05E-20	0.728	0	1.27	0.594	0.014
**OPHN1**	**435**	**199**	**2.08**	**6.15E-18**	**0.713**	**0**	**1.49**	**0.635**	**0.00063**
CNR2	435	199	2.1	7.57E-22	0.737	0	1.26	0.61	0.0046
TFF1	435	199	2.11	6.53E-08	0.634	1.1E-08	0.40	0.646	0.00037
FCGR3B	435	199	2.18	8.35E-09	0.642	9.7E-10	1.96	0.547	0.14
TSPAN1	435	199	2.19	1.09E-12	0.676	1.2E-14	0.48	0.605	0.011
HBD	435	199	2.19	1.1E-10	0.66	5.4E-12	1.85	0.533	0.22
SERPINA6	435	199	2.21	7.81E-14	0.685	6.7E-16	1.37	0.558	0.097
SCUBE2	435	199	2.21	6.05E-09	0.644	1.1E-10	0.31	0.65	0.00033
**S100A12**	**435**	**199**	**2.29**	**9.27E-16**	**0.699**	**0**	**1.79**	**0.58**	**0.033**
TFF3	435	199	2.3	8.39E-09	0.642	7.1E-10	0.34	0.639	0.00097
MEFV	435	199	2.31	4.64E-17	0.708	0	1.31	0.604	0.0074
BCAM	435	199	2.33	4.29E-25	0.756	0	0.68	0.645	0.00055
SERPINA5	435	199	2.39	0.000000871	0.622	0.00000007	0.62	0.607	0.011
BEX1	435	199	2.48	2.39E-10	0.657	1.9E-11	0.39	0.505	0.46
CYP2A6	435	199	2.76	6.73E-16	0.7	0	1.27	0.554	0.12
EEF1A2	435	199	3.24	1.67E-17	0.711	0	0.54	0.542	0.19
CGA	435	199	3.87	1.71E-17	0.71	0	0.92	0.503	0.48
CPB1	435	199	4.8	4.19E-12	0.671	7.4E-14	0.24	0.606	0.014
SMR3B	435	199	5.16	5.71E-11	0.662	6.1E-11	19.55	0.567	0.069
CARTPT	435	199	8.12	8.83E-16	0.699	0	2.20	0.504	0.46
NTS	435	199	14.7	1.24E-08	0.641	3.1E-10	6.72	0.571	0.057
